# Omics-Based Interaction Analysis Reveals Interplay of Chemical Pollutant (Ozone) and Photoradiation (UVSSR) Stressors in Skin Damage

**DOI:** 10.3390/biology14010072

**Published:** 2025-01-14

**Authors:** Hong Zhang, Yiying Dong, Xue Xiao, Xiao Cui, Xuelan Gu

**Affiliations:** Unilever R&D Shanghai, 66 Lin Xin Road, Linkong Economic Development Zone, Shanghai 200335, China; hong-sh.zhang@unilever.com (H.Z.);

**Keywords:** omics-based interaction effect, ozone, ultraviolet, environmental stressors, co-exposure, skin damage, 3D living skin equivalent model

## Abstract

The skin acts as the first line of defense against various environmental stressors, such as solar ultraviolet radiation, visible light, pollution particles and ozone. Simultaneous exposure to different stressors is common in everyday life but has been less studied than exposure to single stressors. Herein, the combined effects of a chemical pollutant (ozone) and a UV radiation stressor (UVSSR) were investigated on a 3D pigmented living skin equivalent model. Our results demonstrate that skin lightness (L* value) was significantly decreased by exposure to either UVSSR or ozone alone and that co-exposure to UVSSR and ozone further exacerbated surface darkness, suggesting that these stressors had a significant joint effect. Conventional differential expression analysis showed that, among exposure groups, co-exposure dysregulated the most genes, followed by ozone and UVSSR alone. Omics-based interaction framework (OBIF) analysis showed that most interactive genes following ozone and UVSSR exposure displayed a cooperative effect and had functions related to the skin barrier; these genes with synergistic effects were enriched in biological pathways such as the chronic inflammatory response and the apoptotic signaling pathway. In summary, exposure to ozone in combination with UVSSR showed a joint effect on UVSSR-induced phenotypic changes in the skin; the underlying mechanism was determined by using transcriptome analysis, showing the additive impacts of ozone on UVSSR-induced skin damage, such as cellular stress and inflammatory responses. These findings shed light on how ozone exacerbates UVSSR damage and indicate that the synergistic response genes identified using OBIF analysis may drive the progression of skin damage induced by chemical/photoradiation stress co-exposure.

## Simple Summary

While skin damage caused by a single environmental stressor, particularly ultraviolet radiation, is well understood, far less is known regarding the exposure to multiple stressors, especially the interplay of multiple stressors, which is crucial for the development of skin protection technologies. In this study, for the first time, we investigated the combined effects of UVSSR and ozone on a 3D pigmented skin model and determined the underlying driver genes associated with the additive effect of ozone on UVSSR-induced skin damage by using an omics-based interaction framework analysis, proposing the vital biological processes contributing to the interplay effect (graphical abstract). The results highlight the importance of adequate protection against UV and ozone, providing a scientific foundation for sunscreen product development.

## 1. Introduction

As the outermost layer of the human body, the skin acts as a shield against external stressors, such as ultraviolet (UV) radiation and pollutants. These stressors damage the skin by affecting its physiology, accelerating aging and even inducing skin diseases [1,2,3]. UV radiation is the most common skin stressor due to daily exposure. Excessive exposure to UV irradiation, including UVB and UVA, results in significant skin damage, such as pigmentary disorders, inflammation and even skin cancer [4]. Among environmental stressors, ground-level ozone is one of the most harmful pollutants to humans. Composed of three oxygen atoms, ozone in the upper atmosphere protects the Earth from UV radiation. However, ground-level ozone is one of the six “criteria air pollutants” described by the Environmental Protection Agency (US EPA) and could be the top issue to address in air pollution control through improvements in pollution control and environmental quality awareness [5]. Ground-level ozone is generated by chemical reactions between oxides of nitrogen and volatile organic compounds (United States Environmental Protection Agency). Exposure to ozone can induce various health problems and compromise skin health [2,6,7,8,9]. For example, ozone was reported to be associated with respiratory diseases [10] in investigations on the association between ozone exposure and hospital admission [11], and the authors of a population-based cohort study suggested that long-term exposure to ozone was positively associated with the probability of pneumonia [12,13].

As the interface between the human body and environmental stressors, the skin is directly exposed to ozone, which has been shown to induce lipid oxidation and result in antioxidant depletion in the stratum corneum [14,15]. Quantitative research has shown that squalene, a skin lipid, might be a major scavenger of ozone at the interface between the air and the human body [15,16,17]. A decrease in squalene levels in the skin after ozone exposure was previously reported [18]. The reaction between ozone and squalene increases levels of volatile products, which may be skin irritants. In vitro studies have also shown that ozone activates the inflammasome, leading to inflammatory skin conditions [19], affects lipid homeostasis, induces histological changes in full-thickness models and affects extracellular matrix integrity [20].

Ozone levels present seasonal variations, with the highest levels in summer and the lowest levels in winter [21]. UV radiation also reaches higher levels in summer than in other seasons. The skin is commonly exposed to multiple environmental stressors in everyday life, and co-exposure to or the synergistic effect of multiple environmental stressors poses significant risks to the skin [22]. For instance, co-exposure to UV and urban particulate matter showed a strong effect on the reduction in the cell proliferation rate [23]. UV damage to keratinocytes could be enhanced by particulate matter (BaP) pre-exposure [24]. Ozone was shown to increase the levels of oxinflammation markers in UV-damaged skin explants [25,26].

With the aim of better understanding the underlying mechanisms of interactions among different stressors, a few studies were carried out based on omics approaches [27,28]. Liu et al. predicted the joint toxicity of particulate matter by incorporating genomic similarity and the dose–response curves of pollutants. The study results provide a comprehensive understanding of the joint toxicity of pollutants, including residual effects. However, the omics calculation part was based on similarities in gene expression. Here, the effects of exposure to UVSSR and ozone alone and to both stressors simultaneously on phenotypic change were investigated in a 3D living skin equivalent model. Subsequently, an omics-based interaction framework approach [29] was adopted to investigate the interaction effect of UV and ozone at the transcriptomic level and identify the driver genes of potential synergistic effects to enrich existing knowledge on the synergistic interplay of UV and ozone in skin damage.

## 2. Materials and Methods

### 2.1. Three-Dimensional Skin Model Challenge

Pigmented living skin equivalent (pLSE) models were purchased from Biocell (MelaKutis^®^, Guangdong, China). Upon arrival, the models were maintained in a recovery medium for 18 h; then, they were transferred to the culture medium. In this study, four testing groups were set up: control, ozone, UVSSR and co-exposure groups. In the control group, the culture medium was refreshed daily. In the ozone group, the pLSE models were exposed to 0.1 ppm ozone for two hours in a pollution chamber (Shanghai Guangpin Equipments Co., Ltd., Shanghai, China); after ozone exposure, the culture medium was refreshed. In the UVSSR group, the pLSE models were irradiated with 100 mJ/cm^2^ solar ultraviolet radiation (UVSSR) by using a solar simulator (Sol-UV-6; Newport, MA, USA); then, the medium was refreshed. In the ozone and UVSSR co-exposure group, the pLSE models were exposed to 0.1 ppm ozone for two hours, followed by treatment with 100 mJ/cm^2^ UVSSR irradiation; after the procedure, the culture medium was refreshed. All treatments were repetitively conducted for three days (Supplementary Figure S1). Samples were collected 24 h after the last treatment for further analysis.

### 2.2. Surface Brightness Measurement

The pLSE models were cut from the insert and placed on white plastic plates with the stratum corneum side up. The L* value, as the indication of lightness in the CIELAB color scale model, was measured with the spectrophotometer CM700 (Konica-Minolta, Tokyo, Japan). There were five replicates in each treatment group. The L* value of each sample was measured three times, and the mean value was used for further analysis.

### 2.3. Statistics Analysis

The statistical differences among the exposure groups were evaluated by using ANOVA, followed by Tukey’s HSD post hoc test. Adjusted *p*-values smaller than 0.05 were considered to indicate significant changes.

### 2.4. Transcriptomic Profiling of pLSE Models

In the transcriptomic study, there were three replicates for each treatment group. The pLSE models were collected 24 h after the last treatment for the RNA-seq study. Total RNA was extracted by using TRIZOL Reagent (Cat#15596-018; Life technologies, Carlsbad, CA, USA); RNA concentration was measured by using a NanoDrop ND-2000, and its integrity was checked by using an Agilent Bioanalyzer 2100 (Agilent technologies, Santa Clara, CA, USA). Total RNA was purified by using an RNAClean XP Kit (Cat A63987; Beckman Coulter, Inc. Kraemer Boulevard Brea, CA, USA) and an RNase-Free DNase Set (Cat#79254; QIAGEN GmBH, Hilden, Germany). RNA sequencing was conducted by Shanghai Biotechnology by using HiSeq2500 (Illumina, CA, USA).

### 2.5. Bioinformatic and Interaction Analyses

Cleaned reads were mapped to the human genome (hg38, Ensemble). Differentially expressed genes (DEGs) for UVSSR against control, ozone against control and co-exposure against control were identified by using EdgeR with a cut-off *p*-value < 0.05 and absolute fold change > 2. Pathway analysis was performed by using Ingenuity Pathway Analysis (IPA; QIAGEN). GO enrichment analysis was conducted by using the R package clusterProfiler (R version 4.2.0).

The interaction effect between the two stressors was predicted by using an omics-based interaction framework (OBIF) [29]. The main, simple main and interaction effects were evaluated based on the full factorial analysis of gene expression. All genes with interaction effects (interaction *p*-value < 0.05) were classified into eight components to characterize the cooperative or competitive effect of the two stressors. There was a cooperative effect on the assessed gene if both stressors regulated gene expression in the same direction; otherwise, there was a competitive effect. Within the cooperative component, there was a concordant effect on the gene if co-exposure regulated gene expression in the same direction as individual exposure; otherwise, there was a discordant effect. Within the competitive component, there was a UVSSR-dominant effect if co-exposure regulated gene expression in the same direction as UVSSR exposure; otherwise, the effect was ozone-dominant.

The synergy score for each gene was calculated as follows:

$${Log}_{2}\left|\frac{{Log}_{2}\;(coexposure)}{{Log}_{2}\;\left(UVSSR\right)+{Log}_{2}\;(ozone)}\right|.$$

A positive score indicated a potential synergistic effect. The synergy score was calculated as the absolute ratio of log_2_ fold change under co-exposure to the sum of log_2_ fold changes under exposure to the two individual stressors.

## 3. Results

### 3.1. Additive Effects of UVSSR and Ozone on Skin Pigmentation

**Figure 1:**
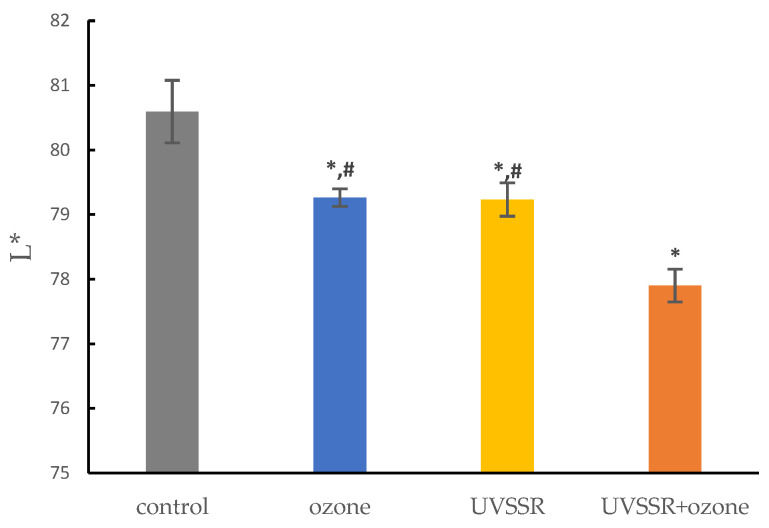
Effects of exposure to UVSSR or ozone alone and to both stressors simultaneously on brightness (L* value). Data are shown as means ± SDs. *: adj *p*-value < 0.05 compared with control. #: adj *p*-value < 0.05 compared with co-exposure to UVSSR and ozone. Statistical differences among exposure groups were evaluated by using ANOVA, followed by Tukey’s HSD post hoc test.

Exposure to a single stressor, UVSSR or ozone, significantly decreased the L* value, with no notable difference between the two groups. Co-exposure to UVSSR and ozone also induced a significant decline in the L* value. Notably, the reduction in L* was significantly greater under co-exposure than under exposure to either UVSSR or ozone alone (Figure 1), suggesting that exposure to a combination of the two stressors can have a joint effect in terms of stress-induced pigmentation.

### 3.2. Common and Divergent Gene Expression Changes Induced by Exposure to Individual Stressors and to Both Stressors Simultaneously

**Figure 2:**
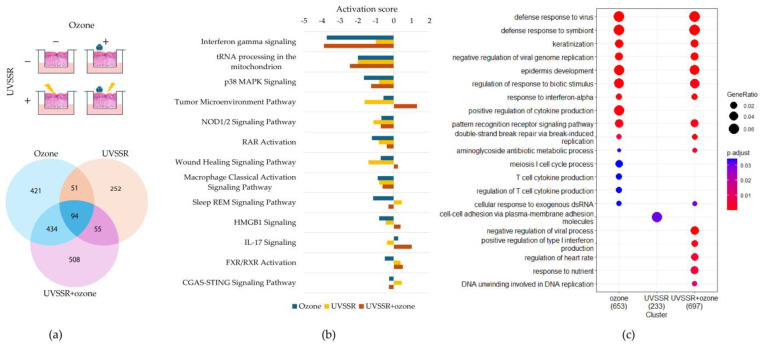
Transcriptomic profiling investigation of pLSE models exposed to individual or combined stressors. (**a**) Overlapping DEGs of any two challenge groups. (**b**) Top pathways of individual exposure regimes ranked by *p*-value predicted with IPA. (**c**) Comparison of biological processes under exposure to individual and combined stressors (cut-offs: *p*-value < 0.05, q-value < 0.05).

Transcriptomic profiling following exposure to UVSSR or ozone alone and to both stressors simultaneously revealed that co-exposure induced more differentially expressed genes than individual stressors (Figure 2a), while there was limited dysregulation in both the UVSSR and ozone groups. About 508 genes were specifically differentially expressed under co-exposure to UVSSR and ozone. The gene expression profile change induced by co-exposure to the two stressors was more similar to that induced by exposure to ozone alone than UVSSR alone (Supplementary Figure S2).

Genes dysregulated by co-exposure were found to be involved in a wide range of pathways (IPA; Supplementary Tables S1–S3), while exposure to single stressors, especially UVSSR, showed limited outcomes. The interferon gamma signaling, p38/MAPK signaling, RAR activation, wound-healing, HMGB1 signaling and IL-17 signaling pathways were all significantly associated with DEGs under single-stressor exposure and co-exposure. Among all significantly involved pathways, the interferon signaling, toll-like receptor signaling, HMGB1 signaling, neuroinflammation signaling and VDR/RXR activation pathways showed high significance (lower *p*-values) and were among the top 10 pathways (Figure 2b). The predicted pathway activity showed that ozone had the strongest impact on the p38 MAPK signaling and RAR activation pathways among all three treatments; UVSSR had the strongest impact on the wound-healing signaling pathway. Finally, co-exposure showed a stronger influence on the interferon gamma and IL-17 signaling pathways than exposure to UVSSR or ozone alone.

The DEGs dysregulated following co-exposure to UVSSR and ozone were predicted to be involved in more than 47 pathways, including the WNT/β-catenin, endothelin-1 and TGF-β signaling pathways. Ozone specifically regulated some inflammatory pathways, such as the IL-10 signaling, DNA damage-induced senescence and melanocyte development and pigmentation signaling pathways. In the melanocyte development and pigmentation signaling pathway, *PAX3* and *SOX10* were upregulated by ozone; these two genes have been reported to act on MITF expression to promote pigmentation [30,31].

The GO enrichment analysis of the top biological processes showed that the co-exposure-regulated processes were more similar to those regulated by exposure to ozone than to UVSRR, as they both regulated keratinization and epidermis development (Figure 2c), while UVSSR exposure in the current model regulated limited biological processes. The specific DEGs under co-exposure (Figure 2a) were associated with keratinocyte differentiation and epidermis development as well (Supplementary Table S4).

### 3.3. Omics-Based Interaction Effect of UVSSR and Ozone

**Figure 3:**
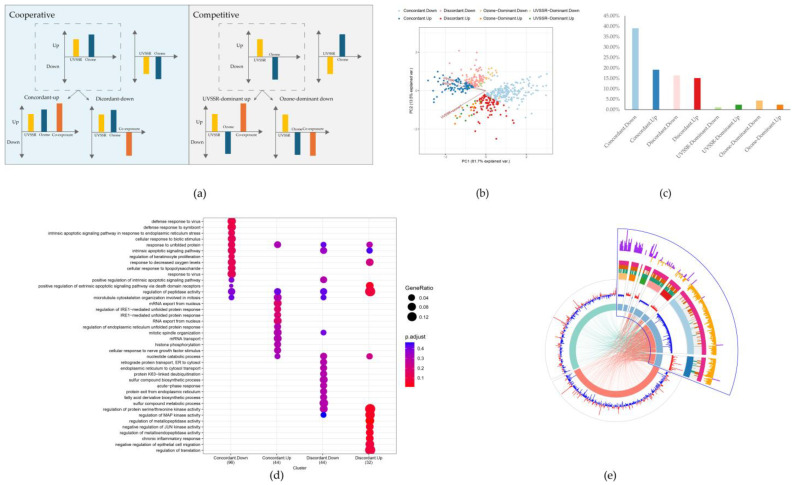
Interaction effects of UVSSR and ozone at the transcriptomic level. (**a**) A scheme of expression profile components. There were eight expression profiles based on the expression change direction, classified as cooperative (same regulation direction for UVSSR and ozone) and competitive (opposite regulation directions for UVSSR and ozone) and further as concordant, discordant, UVSSR-dominant and ozone-dominant. (**b**) Principal component analysis of interaction genes. (**c**) Distribution of interaction genes on eight expression profile components. (**d**) Biological process comparison of cooperative genes based on GO enrichment analysis. (**e**) Visual schematic of interaction genes. Interaction genes with fold change greater than 1.2 are represented by the inner circle (red for upregulation and blue for downregulation); among these, those under co-exposure are highlighted in the right corner with expression profile information (middle circle) and interaction scores (outermost circle, where purple represents a positive interaction score as an indication of synergy and yellow represents a negative interaction score).

The OBIF was adopted to investigate the interaction effect on gene expression exerted by UVSSR and ozone based on the identification of the main and simple main effects of exposure to individual and combined stressors [29]. Further, the expression profile components were identified to determine the competitive/cooperative effects of UVSSR and ozone (Figure 3a). They exerted a significant interaction effect on 548 genes. The interacting gene cluster analysis clearly illustrated that most of the interacting genes (90%) were cooperatively expressed under UVSSR and ozone exposure (Figure 3b). Within the cooperative component, 65% of the genes were concordantly expressed under co-exposure and individual exposure (Figure 3c), including *DDIT3*, *DDIT4, KLF9*, *HOTAIR*, *OVOL2* and *ECM2*. The remaining cooperative genes were discordant, where the expression of genes such as *ALDH1A3*, *APOL2* and *SPINK6* under co-exposure was opposite to that under individual exposure. A limited number of interacting genes were dominantly regulated by either UVSSR or ozone. Ozone dominantly affected more interacting genes than UVSSR. UVSSR showed a dominant impact on genes such as *S100A8*, *CSTB* and *TAB2,* while ozone showed a dominant impact on genes such as *ALDH3A2*, *S100A9*, *APAF1* and *NFKBIA*.

The functional analysis of the genes in each component revealed that these interacting genes were involved in pathways such as the aryl hydrocarbon receptor (AHR) signaling pathway, the fatty acid oxidation pathway and the p38/MARK signaling pathway (Supplementary Table S5). Keratinocyte proliferation-, response to oxygen- and apoptotic processes-related genes were concordantly regulated by exposure to UVSSR or ozone alone and to both stressors simultaneously. Discordant genes were mainly involved in chronic inflammatory response (Figure 3d).

**Figure 4:**
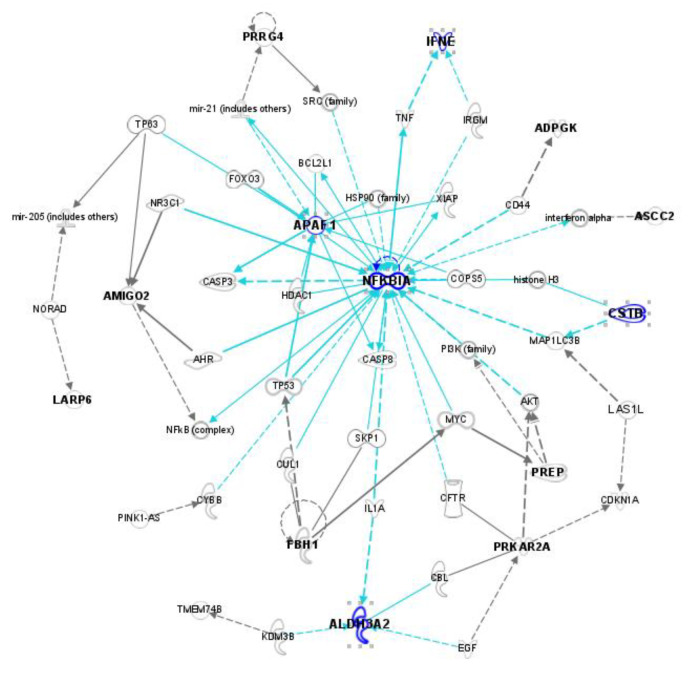
The network of potential synergy genes predicted by using IPA. The network demonstrates the indirect interaction among potential synergy genes (bold). The edges linking NFKBIA, APAF1, ALDH3A2, CSTB and IFNE are highlighted to show their indirect links.

**Figure 5:**
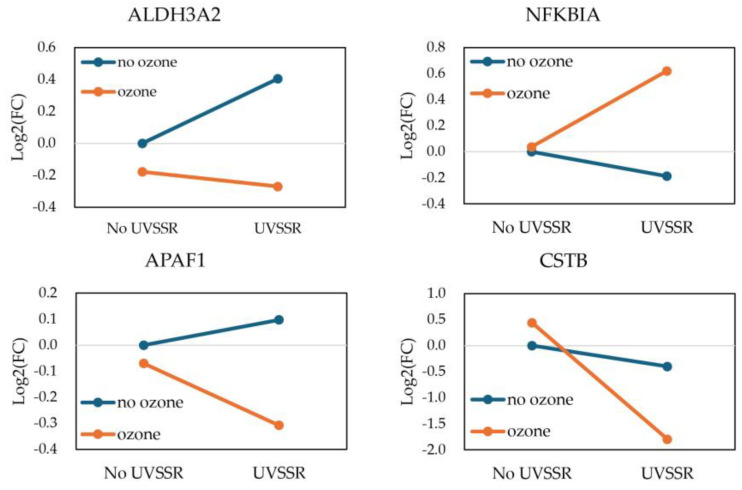
Expression of synergy genes under exposure to individual and combined stressors.

The visual summary highlights interacting genes with fold changes greater than 1.2 under exposure to UVSSR or ozone alone and both stressors simultaneously (Figure 3e). The interaction genes with a positive synergy score and significant expression change (*p*-value < 0.05 and absolute fold change > 1.2 of the main effect) were identified as the potential synergy genes for UVSSR and ozone. In contrast to the interacting genes, most of these potential synergy genes (68.7%) were competitively expressed under UVSSR and ozone, and most of them were dominantly regulated by ozone. These genes were highly predicted to be involved in TWEAK signaling, apoptosis-related pathways and NAD metabolism-related progress based on IPA. The relationships of these potential synergy genes were explored by using an IPA network. *NFKIBIA*, *APAF1*, *ALDH3A2*, *PREP*, *FBH1*, *PRKAR2A*, *LAS1L*, *AMIGO2*, *ADPGF*, *IFNE*, *ASCC2*, *LARP6* and *CSTB* can be linked to each other indirectly (Figure 4). Most of these genes, such as *APAF1*, *NFKIBIA* and *IFNE*, which had high synergy scores, and *ALDH3A2*, were ozone-dominantly regulated (Table 1). *APAF1*, which functions as an orchestrator in the apoptotic pathway [32], was dominantly regulated by ozone. *ALDH3A2*, which plays a vital role in lipid peroxidation, showed a similar result. The expression of *CSTB*, which has been reported to potentially regulate proliferation/differentiation [33] and melanin content [34], was also potentially synergistically regulated by UVSSR and ozone, predominantly by UVSSR. Figure 5 demonstrates how these genes were interactively regulated by UVSSR and ozone. These results indicate that UVSSR and ozone potentially demonstrate a synergistic effect by regulating inflammation, lipid peroxidation and apoptosis, driving multiple biological processes downstream.

## 4. Discussion

In this study, the skin damage induced by UVSSR and ozone individually and the combined damaging effect of co-exposure to these stressors were investigated in living skin equivalent models, and the interplay of UVSSR and ozone in skin damage was determined with OBIF analysis.

Ozone pollution has been gaining increasing attention recently due to its increased levels and harmful effects on human health; it can oxidize skin surface lipids and activate oxidative and inflammatory responses, leading to skin damage. UV exposure can penetrate the dermis or epidermis and induce cell DNA damage, inflammation and pigmentation, but there is limited evidence to support how ozone acts additively to UV in inducing skin darkness. Here, 3D pigmented living skin equivalent models exposed to one or two stressors were leveraged to mimic real-life exposure. In our model, both UVSSR and ozone induced skin pigmentation, and co-exposure to UVSSR and ozone had a clear combined effect on skin darkening. The change in L* due to co-exposure was approximately equal to the sum of the change induced by UVSSR and ozone, suggesting a joint effect of UVSSR and ozone on the acquired hyperpigmentation.

The differential expression analysis results show that exposure to ozone alone and in combination with UVSSR induced more gene expression changes than UVSSR exposure. The differentially expressed genes were found to be significantly involved in the interferon signaling pathway, the HMGB1 signaling pathway and glutamine degradation. These pathways have been reported to act on pigmentation processes and potentially as underlying molecular functions of skin pigmentation regulation under stress. UVSSR and ozone only shared a small number of differentially expressed genes (Figure 2a), indicating a clear, different expression impact on skin damage. The functional analysis showed that ozone regulated skin keratinization and epidermis development processes, while UVSSR regulated genes involved in AHR pathways and cell–cell adhesion. The divergence between UVSSR and ozone at both gene and functional levels suggests that these stressors damage the skin through different pathways and could lead to a combined effect on skin damage. At the individual gene expression level, late cornified envelope genes, MMPs and oxidation-related genes were differentially expressed under co-exposure, but their expression was not consistent, driving us to holistically explore their function and potential interaction effect at the omics level.

Repeated co-exposure to UVSSR at 100 mJ/cm^2^ and ozone at 0.1 ppm regulated the gene expression profiles, and the biological processes displayed similar patterns under ozone alone and co-exposure, unlike under UVSSR. Previous studies showed that ozone at 0.25 ppm and UV at 200 mJ/cm^2^ had an additive effect on skin damage, particularly oxinflammation damage. Ozone, as a strong oxidizing agent, can cause lipid peroxidation in the upper epidermal layers; although ozone is unable to penetrate the skin, the oxidative stress caused by ozone compromises the skin barrier function and makes the skin more susceptible to the exposome, such as UV exposure. The pathway analysis showed that inflammatory pathways such as the IL-17 signaling pathway were significantly associated with the genes dysregulated by exposure to individual or combined stressors. IL-17 is well known for its function in skin disorders such as psoriasis [35], and a transcriptomic study showed that it was a key mediator for airway inflammation induced by ozone [36]. Therefore, the involvement of this pathway in our study suggests that the compromised skin barrier makes the skin more susceptible to inflammation, leading to phenotypic change, such as lightness change. For the first time, this finding helps to understand the cellular mechanisms of ozone-induced skin pigmentation and provides new insights into the additive effect of ozone on the UV stress response in the skin.

The OBIF method was employed to investigate the interaction between two treatments and determine the molecular drivers of synergy with a full factorial analysis of the gene/protein expression data from single versus dual treatments. The method was leveraged here to identify (1) the genes on which UVSSR and ozone have an interaction effect, (2) the cooperative/competitive genes under UVSSR and ozone, and (3) potential synergy mechanisms. As expected, UVSSR and ozone had a cooperative effect on gene expression change. The cooperative interaction genes, such as KLF9, ECM2, OVOL2 and SPINK6, are linked to skin barrier functions, which suggests a potential additive effect on skin barrier damage of UVSSR and ozone. On the other hand, the synergy genes, such as ALDH3A2, APAF1 and IFNE, were more ozone-dominantly regulated. ALDH3A2 is involved in the conversion of aliphatic aldehydes into fatty acids for the main skin barrier. APAF1 is involved in apoptosis; it binds to cytochrome c to form the apoptosome and plays a role in maintaining cell homeostasis.

The IPA network and pathway analysis showed that these synergy genes are related to apoptosis, lipid metabolism, inflammatory response, etc. Combined with the biological processes associated with the dysregulated genes under co-exposure, these results suggest that UVSSR and ozone play vital roles in skin damage, exerting effects such as the dysregulation of keratinization, epidermis development and lipid metabolism, cell apoptosis and inflammation.

Synergy is commonly identified by using the Loewe model or Bliss model [37] based on dose–response data. Synergy prediction using big omics data is mainly based on gene expression similarity or machine learning methods, providing a limited understanding of the underlying mechanism [38,39,40]. Here, we applied the OBIF method from another perspective to identify genes with interaction and potential synergy effects by incorporating the main, simple main and interaction effects based on full factorial analysis. This method allowed us to obtain more explainable insights into the interplay between UVSSR and ozone and identify the driver genes for synergistic effects. However, more wet experiments are required to validate the predicted synergy drivers and prioritize key biomarkers for skin protection assessment. The interaction analysis results are compatible with those of the conventional analysis since most of the synergy drivers were ozone-dominantly expressed, and the co-exposure-regulated gene expression profile was much more similar to that of ozone. This finding emphasizes the importance of chronic ozone in skin damage induction. Technological development to protect skin from ozone exposure rather than UVSSR alone is necessary to achieve better efficacy in protection from stressors.

The focus of this study was exploring the interaction effect between two different stressors from the perspective of whole-transcriptomic gene expression, but with limitations on biomarker validation. It is well known that UV irradiation can induce DNA damage, oxidative damage, ROS generation and inflammation and affect the melanogenesis pathway, resulting in phenotypical skin pigmentation. On the other hand, there is limited research on pigmentation induced by ozone, although abundant studies have demonstrated its effect on oxidation and inflammasome activation. Here, we present the first study to demonstrate the effect of this stressor on pigmentation in a 3D skin model, and the results show that ozone exposure upregulated *SOX10* and *PAX3*, activating MITF expression to promote skin pigmentation. Further, this study also showed the additive effect of ozone on UVSSR. However, the molecular phenotype was not investigated. Nevertheless, the advanced interaction analysis results suggest that the regulation of genes related to apoptosis, inflammatory response and lipid peroxidation by these stressors might be key to their additive effect on pigmentation. In the future, comprehensive research is needed to consolidate the detailed mechanism of co-exposure and single-stressor exposure. In addition, it is also worth investigating various dosage ratios of the two stressors to determine different mechanisms for different co-exposure ratios.

## 5. Conclusions

Co-exposure to UVSSR and ozone resulted in pigmentation in a 3D skin model. Whole-transcriptome profiling demonstrated that co-exposure-induced gene and function dysregulation was mainly driven by ozone. Interaction effect analysis indicated that UVSSR and ozone have a cooperative effect on the skin barrier, and synergy drivers were identified, shedding light on the mechanism of potential joint effects on skin damage.

## Figures and Tables

**Table 1:** Synergy scores of genes.

Gene	Component	Synergy Score
*PREP*	Concordant	1.00
*ADPGK*	Concordant	2.04
*FBH1*	Discordant	0.95
*PRKAR2A*	Discordant	1.02
*ALDH3A2*	Ozone-dominant	0.26
*ASCC2*	Ozone-dominant	1.28
*LAS1L*	Ozone-dominant	1.67
*APAF1*	Ozone-dominant	3.51
*AMIGO2*	Ozone-dominant	1.60
*IFNE*	Ozone-dominant	2.98
*NFKBIA*	Ozone-dominant	2.04
*LARP6*	Ozone-dominant	1.29
*CSTB*	UVSSR-dominant	0.85

## Data Availability

The present study’s data are available upon request to the corresponding author due to the privacy policy.

## Supplementary Materials

The following supporting information can be downloaded at https://www.mdpi.com/article/10.3390/biology14010072/s1: Figure S1: Graphic illustration of experiment design; Figure S2: Gene expression similarity; Tables S1–S3: Pathways associated with DEGs; Table S4: GO items associated with specific DEGs under UVSSR and ozone co-exposure; Table S5: Pathways associated with interaction genes.
