# Supplementary material for: Omics-Based Interaction Analysis Reveals Interplay of Chemical Pollutant (Ozone) and Photoradiation (UVSSR) Stressors in Skin Damage
Source: Biology (Basel). 2025 Jan 14;14(1):72. doi: 10.3390/biology14010072 (PMC11759167; doi:10.3390/biology14010072)
Supplement: Supplementary file 1 [file biology-14-00072-s001.zip › biology-3376498-supplementary.pdf]

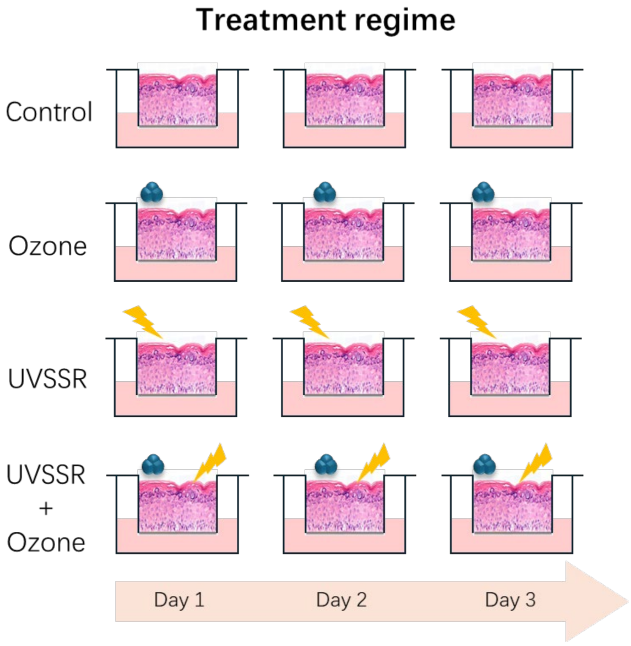

Figure S1: Graphic scheme for the experimental procedure.

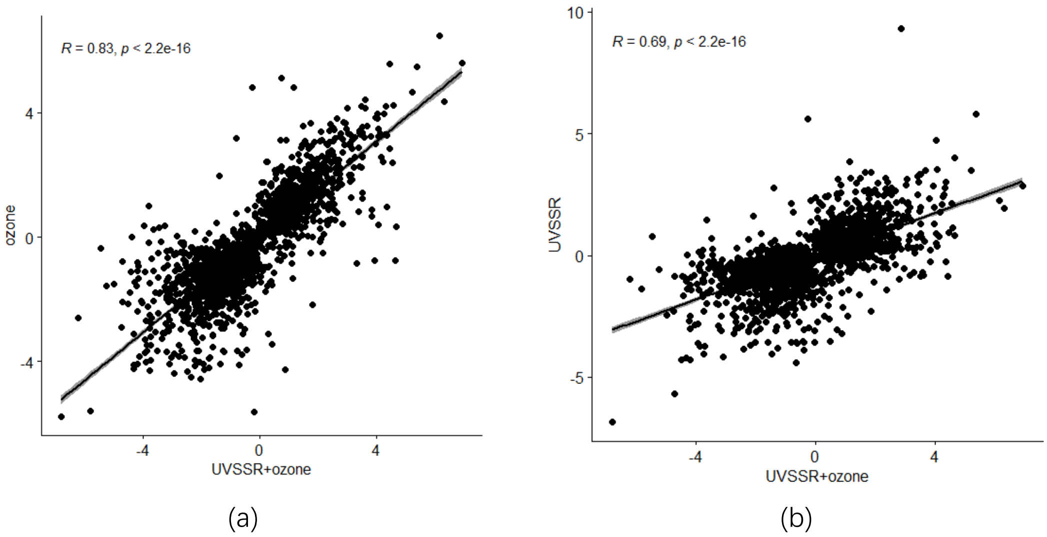

Figure S2: Gene expression similarity between any two treatments. (a): Gene expression similarity between ozone and co-exposure. (b): Gene expression similarity between UVSSR and co-exposure.
